# “Do-it-yourself *in vitro* vasculature that recapitulates *in vivo* geometries for investigating endothelial-blood cell interactions”

**DOI:** 10.1038/srep12401

**Published:** 2015-07-23

**Authors:** Robert G. Mannino, David R. Myers, Byungwook Ahn, Yichen Wang, Margo Rollins, Hope Gole, Angela S. Lin, Robert E. Guldberg, Don P. Giddens, Lucas H. Timmins, Wilbur A. Lam

**Affiliations:** 1 Wallace H. Coulter Department of Biomedical Engineering, Georgia Institute of Technology and Emory University; 2 Emory University School of Medicine, Department of Pediatrics, Division of Pediatric Hematology/Oncology; 3 Childrens Healthcare of Atlanta, Aflac Cancer & Blood Disorders Center; 4 Institute of Electronics and Nanotechnology, Georgia Institute of Technology, Atlanta, GA, United States; 5 Emory University School of Medicine, Division of Cardiology, Department of Medicine; 6 Petit Institute for Bioengineering and Biosciences, Georgia Institute of Technology; 7 School of Mechanical Engineering, Georgia Institute of Technology

## Abstract

Investigating biophysical cellular interactions in the circulation currently requires choosing between *in vivo* models, which are difficult to interpret due in part to the hemodynamic and geometric complexities of the vasculature; or *in vitro* systems, which suffer from non-physiologic assumptions and/or require specialized microfabrication facilities and expertise. To bridge that gap, we developed an *in vitro* “do-it-yourself” perfusable vasculature model that recapitulates *in vivo* geometries, such as aneurysms, stenoses, and bifurcations, and supports endothelial cell culture. These inexpensive, disposable devices can be created rapidly (<2 hours) with high precision and repeatability, using standard off-the-shelf laboratory supplies. Using these “endothelialized” systems, we demonstrate that spatial variation in vascular cell adhesion molecule (VCAM-1) expression correlates with the wall shear stress patterns of vascular geometries. We further observe that the presence of endothelial cells in stenoses reduces platelet adhesion but increases sickle cell disease (SCD) red blood cell (RBC) adhesion in bifurcations. Overall, our method enables researchers from all disciplines to study cellular interactions in physiologically relevant, yet simple-to-make, *in vitro* vasculature models.

The pathophysiology of various hematologic and vascular diseases such as sickle cell disease, malaria, myocardial infarction, and stroke involve pathologic biophysical cellular interactions among different blood cell subpopulations and the endothelium^1^,^2^,^3^,^4^. These interactions are mediated by the hemodynamic forces of the circulation, which, in turn, are dictated by local vessel geometry. For example, platelet activation and aggregation occurs most readily in high shear conditions of a stenotic arterial lesion^4^,^5^. Therefore, a comprehensive and quantitative understanding of disease pathophysiology requires studying these pathologic biophysical cellular interactions in the context of vascular geometries. This is infeasible, however, with current animal models as neither flow nor vessel geometry can be tightly controlled.

*In vitro* flow-based models have provided valuable insight but are often either too simple to accurately reflect physiologic conditions or too complex as to preclude widespread use. For example, standard *in vitro* models often employ cone-and-plate viscometry to induce shear forces on endothelial monolayers, but this neglects vascular geometry and the resulting complex flow patterns (e.g., secondary flow)^6^. Microfluidic devices fabricated with photolithographic techniques have advanced our understanding of flow-based cell-cell interactions^7^,^8^, but these techniques require access to advanced tools and microfabrication facilities^9^ and are confounded by non-physiological flows resulting from the rectangular cross section of microchannels^10^. 3D printing has been used to develop *in vitro* vasculature models that result in improvements upon typical *in vitro* systems^11^. However, these systems cannot readily recapitulate vascular geometries relevant to disease pathophysiology and are not inexpensively available to the typical biomedical researcher^11^. Macroscale models to bridge *in vivo* and *in vitro* studies have focused on creating molds derived from living tissue^12^,^13^ but require numerous tissue samples and castings and suffer from reduced device yield due to experimental complexity.

Our simple 3-dimensional “endothelial-ized” *in vitro* vasculature addresses the clear need for an *in vitro* model that sufficiently and accurately recapitulates the geometries and cellular environment of blood vessels *in vivo*, as well as the mechanical environments (as confirmed by our computational flow dynamics (CFD) modeling)^14^,^15^. The cylindrical microchannels of our devices support the culture of various types of endothelial cells (Supplemental Fig. 1) and are easily modified to create clinically relevant vascular geometries including stenoses, aneurysms, and bifurcations. This “do-it-yourself” (DIY) vasculature technique requires no microfabrication skills or equipment and is broadly accessible, as inexpensive, common off-the-shelf materials are used to produce precise, reproducible, tunable vascular geometries that induce predictable physiologic alterations in wall shear stress (WSS)^16^.

Our unique and simple DIY method is ideal for dissecting the real-time biophysical blood cell-endothelial interactions in regions where abrupt changes in the vascular geometry induce complex local hemodynamic conditions that are relevant to disease pathophysiology. We explored the effect that geometry and spatial differences in WSS alone have on endothelial cell vascular cell adhesion molecule-1 (VCAM-1) expression as well as endothelial interactions with blood cells (e.g. platelets, sickle cell red blood cells). Our simple, accessible fabrication technique that allows for the recapitulation of vascular geometries enables researchers of all disciplines to quantitatively study the effects of locally varying shear on the vascular endothelium in a way never before possible.

## Results

### The DIY “endothelialized” *in vitro* vasculature with physiologically relevant geometries is broadly accessible, robust, and precise

**Figure 1:**
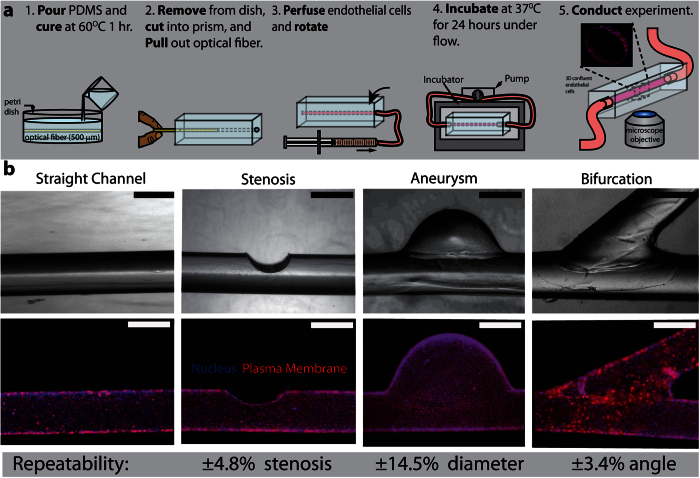
Using commonly available off-the-shelf lab supplies, “do-it yourself” (DIY) vasculature models can be created and cultured with confluent layer of endothelial cells. **a**) Fabrication process of the “do-it-yourself” endothelialized vasculature model. Inset in step 5 shows a confocal image of a straight fully endothelialized channel. Drawings prepared by D.R.M and R.G.M. **b**) Representative bright field (top) and epifluorescence (bottom) images of multiple vascular geometries including a straight channel, stenosis, aneurysm, and bifurcation. Repeatability is the standard deviation of the dimension of interest for each geometry (n = 5). Plasma membrane (Red) and cell nuclei (Blue) are fluorescently stained. Scale bar is 500 μm.

The rapid and simple fabrication method presented here enables researchers of all disciplines to investigate the effects of local alterations in vascular geometry on endothelial function and cell-cell interactions under physiologic flow conditions. Casting PDMS around a PMMA optical fiber creates a hollow, circular channel that can be used as a flow chamber (Fig. 1a). Compared to materials such as metal wires, the low cost, high precision, and extreme smoothness of the PMMA optical fiber provides vastly superior optical properties in the resulting transparent PDMS channels. Devices were seeded with constant rotation to produce an even, confluent monolayer along the entire inner 3D surface of the channel. The seeding process produced confluent endothelial cell monolayers along the inside of the flow chamber using HUVECS (Fig. 1a), as well as HAECs (Supplemental Fig. 1a) and HMVECs (Supplemental Figure 1E). These endothelial monolayers remain functional over a period of 24 hours, as measured by vascular endothelial (VE)-cadherin expression at the cell-cell junctions (Supplemental Fig. 2A) and nitric oxide production (Supplemental Fig. 2B). VE-cadherin maintains barrier function while nitric oxide production is necessary to modulate blood vessel diameter^17^,^18^. Our resulting vasculature model leverages the benefits of microfluidics (e.g. small sample volumes, tightly controlled experimental conditions, ease of imaging), while eliminating its detrimental aspects (e.g. high cost, time-consuming fabrication process involving access to cleanroom equipment and facilities, and specialized training)^19^. In addition, compared to standard microfluidic systems, the microchannels fabricated with this method have rounded lumens, which are more representative of the vascular anatomy than are typical, square-shaped, planar, *in vitro* vascular microfluidic models^10^,^20^.

To fabricate vascular geometries, we subtract material from the straight fiber to create stenoses, add material to create aneurysms, and join fibers together to create bifurcations. To create a stenosis-shaped flow chamber, fine grit sand paper wrapped around a razor blade creates a small, precise notch in the optical fiber prior to casting in PDMS (Fig. 1b, Stenosis). The grit number of the sandpaper determines how smooth the notch (stenosis) is, and the number of sanding strokes determines the degree of stenosis. To create an aneurysm-shaped structure, a drop of wax is placed on a strand of optical fiber prior to casting in PDMS (Fig. 1b, Aneurysm). Sucrose based wax is easily dissolved with water, leaving a dome shaped aneurysm within the channel after the optical fiber strand has been removed and the sugar dissolved. Similarly, bifurcations were created by gluing two fibers together using unique PMMA-dichloromethane solvent glue and annealing the combined structure to maximize smoothness (Fig. 1b, Bifurcation). The solvent glue wicks up the length of the optical fiber strands, creating a seamless transition from glue to fiber, while simultaneously depositing PMMA in gaps between the 2 strands of optical fiber. This results in a smooth channel after the welded optical strands are extracted. The solvent weld breaks apart upon removal, facilitating its extraction and leaving the surrounding PDMS undamaged.

Given the high precision of the PMMA optical fiber, the diameter and shape of the straight sections of each device is highly controlled and repeatable. The technique is broadly accessible as it was designed to be assembled by hand rather than using complex equipment. While some inconsistencies arise within these device geometries due to the reliance on manual dexterity, with a practiced hand, these inconsistencies are negligible. These inconsistencies can be further negated using a variety of device-specific techniques. Placing the optical fiber between two glass slides of known thickness during the sanding procedure required to create a stenosis creates a barrier that stops the sanding at a desired height (corresponding to percent stenosis). In addition, placing the transparent optical fiber and bifurcation assembly tool over a sheet of paper printed with computer-designed angles allows you to reproduce desired bifurcation angles. Using these techniques, stenosis fabrication is accurate to within ±4.8% stenosis, aneurysm radius is accurate to within ±14.5%, and bifurcation angle is accurate to within ±3.4^o^.

### Complex wall shear stress conditions are physiologic and easily tunable by changing geometries

**Figure 2:**
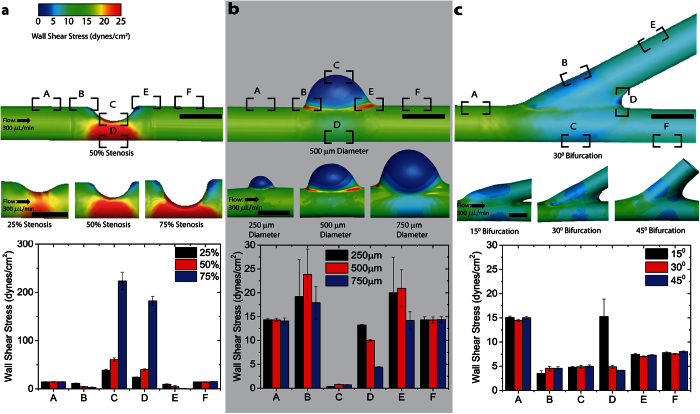
Creating variations in the geometry size enables control over the wall shear stress as shown by computational fluidic dynamics analysis. Computational fluid dynamics analysis of wall shear stress within channels with varying **a**) degrees of stenoses, **b**) diameters of aneurysms, and **c**) angles of bifurcations. Physiologic ranges of wall shear stress are altered locally as vascular geometry is modulated. Bar graphs represent the mean wall shear stress of upwards of 100 nodes within each region of interest (i.e., spatial location where governing equations are solved in the computational domain); error bars represent standard deviation. An ANOVA (alpha = 0.05) determined that regions of interest within each geometry experience complex and significantly different wall shear stress (p < 0.000001). Post-hoc two-tailed t-tests (*P < 0.05) revealed significant differences in wall shear stress as the geometry size changes. Scale bar is 500 μm.

CFD simulations were performed to ensure that the endothelial cells cultured within each type of vascular geometry are exposed to physiological WSS values^16^. We observed that the local WSS changes due to geometric variations and alterations in flow rate can be easily calculated in multiple channel locations (Fig. 2, Supplemental Fig. 3). This established that our technique yields highly controlled and precise *in vitro* vasculature that recapitulates the biophysical and changing hemodynamic environment of the *in vivo* vasculature. Each model features geometrically confined regions of high and low shear stress that consistently appear in the same location on all models (Fig. 2). The size and magnitude of these high and low shear stress regions may be tailored by simply changing the device geometry (Fig. 2). For example, percent stenosis determines the maximum wall shear stress, ranging from approximately 40–220 dynes/cm^2^, and at maximum stenosis, a 5.5 fold difference between the high and low shear stress regions is achieved (Fig. 2a). Similar trends were observed in alternate geometries (Fig. 2b,c).

### Variation in VCAM-1 expression correlates with localized changes in wall shear stress due to alterations in vascular geometry

Vascular cell adhesion molecule 1 (VCAM-1) is an adhesion molecule expressed on endothelial cells that is integral to leukocyte transmigration and plays a key role in the inflammatory response of endothelial cells^21^. Previous *in vitro* studies have found that VCAM-1 is either not affected by steady shear stress or is downregulated over time^21^,^22^. Similarly, oscillatory flow conditions *in vitro* result in increased VCAM-1 expression, which gradually decreases to baseline, typically over 24 hours^21^,^23^. In contrast, *in vivo* studies indicate that VCAM-1 is consistently upregulated in areas of estimated lower WSS^24^. Unlike previous *in vitro* systems, our system shows a high level of sustained VCAM-1 expression that changes with the local channel geometry and therefore WSS, accurately recapitulating the *in vivo* environment.

**Figure 3:**
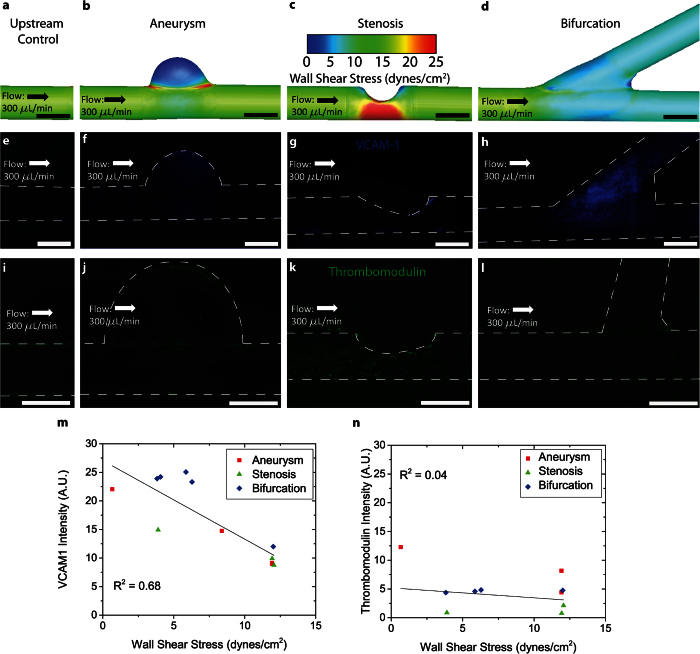
Wall shear stress regulates endothelial expression of VCAM1 in the “do-it-yourself” *in vitro* vasculature, while thrombomodulin expression is not altered. CFD models calculate the wall shear stress (dynes/cm^2^) in **a**) upstream control, **b**) aneurysm, **c**) stenosis, and **d**) bifurcation. **e**–**h**) The locations of highest levels of VCAM1 expression correspond to the areas of lowest WSS in all vascular geometries. **i**–**l**) Wall shear stress does not mediate thrombomodulin expression in the DIY *in vitro* vasculature. **m**–**n**) A custom image analysis script (MATLAB) quantifies the expression of VCAM1 and thrombomodulin. Each device spans a range of shear conditions, and a linear correlation with shear is present in the case of VCAM but not thrombomodulin. Scale bar is 500 μm.

Using CFD techniques, we quantified the WSS experienced by endothelial cells at various locations within our devices (Fig. 3a–d). These results allow us to compare the spatial expression of VCAM-1 with the changes in WSS caused by geometric alterations in blood vessels. Visually, there is a correlation between VCAM-1 expression on endothelial cells (Fig. 3e–h) and areas of low shear stress (Fig. 3a–c). Using CFD modeling and our VCAM-1 data, we were able to correlate WSS values at different locations with VCAM-1 expression, highlighting the quantitative capabilities of our system (Fig. 3m).

After confirming the validity of this model with VCAM-1 expression, we assessed whether other pertinent endothelial signaling pathways unrelated to VCAM-1 are also mediated by localized changes in WSS due to vascular geometry. As such, we studied thrombomodulin, an integral component of the endothelial cell’s anti-thrombotic capabilities, to determine whether endothelial expression of this membrane protein is mediated by local changes in WSS. While one study reported increased thrombomodulin expression in response to elevated shear^25^, another reported a transient increase followed by a significant decrease in thrombomodulin expression in response to increased shear^26^. In our systems, we did not find a thrombomodulin response to locally varying shear stress (Fig. 3i–l,n). The preferential upregulation of VCAM-1 over thrombomodulin indicates that our VCAM-1 results were not due to system artifacts, and further highlights the utility of our system. We are able to recapitulate an *in vivo* vascular environment and correlate areas of lower shear stress to areas of increased adhesion molecule expression. With these results, we have successfully bridged *in vitro* data with an *in vivo* environment in order to highlight the relationship between local changes in WSS and endothelial dysfunction.

### Endothelial cells downregulate platelet adhesion in stenoses

**Figure 4:**
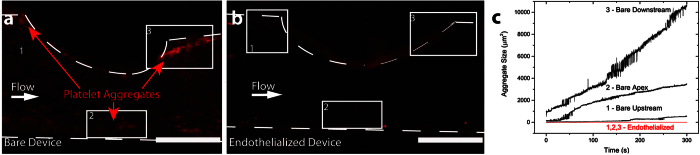
In vascular geometries previously shown to induce platelet aggregation, the presence of cultured endothelial cells attenuates this process, highlighting the need for endothelialized *in vitro* vascular models. **a**) Platelets preferentially aggregate in a “bare” non-endothelialized stenosis at the distal end. **b**) No platelet aggregation is observed when the device is endothelialized. **c**) A custom image analysis script (MATLAB) quantifies the size of platelet aggregates in a DIY stenosis model. Traces 1–3 correspond to total platelet aggregates in boxes 1–3 shown in a–b. Scale bar is 500 μm.

Given the changes in molecular expression, we explored the effects of geometry-mediated WSS changes on blood cell-endothelial cell interactions, focusing on shear-mediated platelet activation as an initial proof of concept. Platelets have been shown to activate in response to shear stress alone, spontaneously and preferentially forming aggregates at the distal end of a microfluidic stenosis over time^27^. We have recapitulated this effect by creating a similar vascular stenosis model with a round lumen that results in higher WSS at the stenosis in a “bare” channel without cultured endothelial cells (Fig. 4a–c). We then repeated this experiment using an identical system, with the exception that a confluent endothelial monolayer was cultured within the stenosis model (Fig. 4b,c). This resulted in a dramatic decrease in platelet aggregation throughout the device, regardless of location within the stenosis or local shear environment. This likely occurs because the vascular endothelium plays an active role in preventing vessel occlusion and platelet adhesion/aggregation by expressing thrombosis inhibitors such as prostacyclin^17^. On the other hand, the endothelium is also capable of enhancing coagulation through the release of ultra-large Von Willebrand Factor (ULVWF), which is involved in shear-activated platelet aggregation^28^. Consistent with previous research, ULVWF^29^ preferentially forms in the high shear region of the vascular stenosis when stimulated with histamine (Supplemental Fig. 4). These results involving blood cell interactions within our DIY vasculature highlight how our *in vitro* system accurately recapitulates key aspects of vascular function and geometry *in vivo*.

### *In vitro* vascular geometries increase endothelial-sickle cell disease erythrocyte adhesion

As pathologic endothelial-erythrocyte adhesion is an integral aspect of sickle cell disease (SCD) pathophysiology, we sought to determine how vascular geometries influence this phenomenon. Previous studies have demonstrated that SCD erythrocytes have greater adherence to the endothelium than healthy erythrocytes do under static conditions^30^ and that this adhesion strongly correlates with clinical symptoms^31^. Studies of SCD erythrocytes using *in vitro* flow chambers^32^ and *ex vivo* rat microvasculature^4^ subsequently confirmed an increase in SCD erythrocyte adhesion under physiological flow conditions.

**Figure 5:**
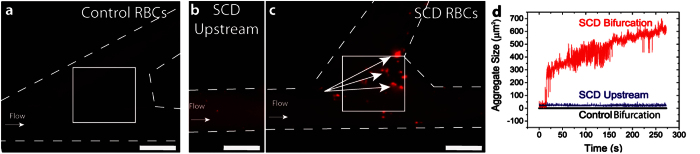
Sickle cell disease (SCD) red blood cells (RBCs) adhere and aggregate at endothelialized bifurcations. **a**) Control RBCs do not form aggregates in a vascular bifurcation after 275 seconds of flow. **b**) Relatively few SCD RBCs form small aggregates in the straight channels in the *in vitro* vasculature upstream of the bifurcation. **c**) SCD RBCs form large aggregates (arrows) at vascular bifurcations. **d**) A custom image analysis script (LabVIEW) quantifies the size of RBC aggregates in a region of interest (box) in a DIY bifurcation model. Control bifurcation corresponds to the box in **a**); SCD upstream refers to RBC aggregation in **b**) (box not shown for clarity); SCD bifurcation corresponds to the box in **c**). Scale bar is 500 μm.

Here we show that a simple geometric bifurcation lined with endothelial cells is capable of inducing massive SCD erythrocyte aggregation, with aggregates covering 6 times (3%) the endothelial surface than previously reported in endothelial-lined flow chambers^32^ (Fig. 5). In fact, previously reported values are a gross underestimate as they represent a 2-dimensional estimate of 3-dimensional RBC aggregates. This highlights the need for studies in bifurcation models, as traditional *in vitro* studies cannot detect a significant portion of aggregation and adhesion events. Surprisingly, although stimulating endothelial cells with the inflammatory cytokine tumor necrosis factor – alpha (TNF-α) did expectedly lead to increased adhesion of SCD RBCs as compared to control conditions (Supplemental Fig. 6), TNF-α endothelial stimulation did not lead to increased SCD RBC adhesion as compared to the unstimulated case, which suggests that SCD RBCs alone are sufficient to cause this phenomenon.

Bifurcations create unique, disturbed flow fields that include recirculation zones^22^,^33^,^34^. Such disturbed flow fields are known to cause endothelial cell changes and the upregulation of adhesion markers^35^. Bifurcations have been specifically linked to SCD stroke: a post-mortem analysis of a sickle cell stroke patient found intimal proliferation, or fibroplasia, localized to bifurcations^36^. The low shear environment of bifurcations is supportive of red cell aggregation, as seen in simulations of post capillary venules^37^ *in vivo*^38^. Hence, while straight channels do not induce aggregations (as shown in previous studies), the unique geometry of the bifurcation induces both endothelial adhesion molecule upregulation (Fig. 3) and alters fluid forces that synergize to create adherent red cell aggregates. Given its flexibility and ease of use, this system offers the unique opportunity to examine a myriad of other pathological conditions such as leukocyte adhesion in cardiovascular disease and the role of leukocytes in sickle cell disease^39^,^40^, which have been observed *in vivo* to aggregate in bifurcations under hypoxic conditions^41^.

## Discussion

Our DIY endothelialized vasculature provides unique insight into the molecular, biophysical, and cellular interactions that occur in the circulation, combining the benefits of reductionist *in vitro* experiments with *in vivo* geometric environments. Our results show that variations in vascular geometry are sufficient to cause physiological changes in the endothelium at the molecular, cellular, and pathological levels due to local changes in WSS. Furthermore, we show that simply altering geometry leads to spatial heterogeneities in WSS values within aneurysms, stenoses, and bifurcations, which differentially affect the inflammatory phenotype of endothelial cells, as measured by VCAM-1 expression. This system meets the current need for a robust *in vitro* vascular model system that can be used to complement current *in vivo* animal models and further dissect the underlying biophysical mechanisms of pathological cell-cell interactions in various diseases. Importantly, the DIY endothelialized vasculature can be fabricated using common, off-the-shelf materials at a fraction of the cost of current *in vitro* techniques and obviates the need for specialized engineering expertise or specialized microfabrication cleanroom facilities, providing researchers of all disciplines newfound access to flow based *in vitro* vascular models.

The findings using the DIY *in vitro* vasculature have clear clinical relevance. Spatial gradients in the WSS experienced by the vascular endothelium have been hypothesized to alter the rate of aneurysm expansion^42^ and have been shown to alter the progression rate of coronary artery disease^43^. With our system, we have experimentally demonstrated how slight geometric alterations in and of themselves cause differential VCAM-1 expression, which correlates with aneurysm expansion^44^. We have demonstrated the ability of our device to recapitulate complex cellular-endothelial interactions with our platelet aggregation experiments. We show that a more physiological environment, created by seeding the rounded channels with a monolayer of endothelial cells, alters and inhibits the spatial distribution of platelet aggregates in vascular stenoses. Finally, we show that introducing regions of disturbed flow via vascular bifurcation increases SCD RBC adhesion and aggregation at the bifurcation branch point. While this is expected from a fluid mechanics perspective, this singular experimental finding yields valuable insight into the pathophysiology of vaso-occlusion in SCD. Additionally, this technique allows the user to easily manipulate the optical fibers into different shapes with different degrees of curvature to create more complex geometries. These simple alterations make this technique a valuable tool for studying endothelial cell - blood cell interactions in angiogenesis and cancer^45^. Overall, these data indicate that the proposed DIY methodology presented here can recapitulate vascular geometries to allow for the dissection of complex biophysical interactions among endothelial cells, blood cells, and soluble factors that were previously technically infeasible to study *in vitro*.

It is important to note that all technologies have limitations, and ours is no exception. The devices are limited in diameter: removing fibers with diameters below 500 μm proved challenging because the fiber tends to snap due to high frictional forces and low ultimate strengths found in high surface area to volume ratio geometries. In addition, devices need to be seeded under constant rotation to avoid preferential seeding within the device. This DIY model of the vasculature represents an easily accessible technique that enables researchers of all disciplines to bridge the gap between *in vitro* and *in vivo* models of molecular and pathological cell-cell interactions in the vasculature. We demonstrate that recapitulating complex *in vivo* vascular geometries is vital to understanding blood cell-endothelial interactions in healthy and disease states.

## Methods

### Fabrication of *in vitro* DIY vasculature

Standard channel – a ~10 cm long strand of 500 μm diameter polymethyl methacrylate (PMMA) optical fiber was placed atop a ~1 mm thick layer of cured PDMS (Dow Corning, Midland, MI) in a 150 mm petri dish and covered with an uncured layer of PDMS of ~1 mm thickness. The second layer of PDMS is then cured. The optical fiber, encased in cured PDMS, was extracted from the petri dish and cut 1 cm from each end. The optical fiber was removed, leaving a microchannel in the cured PDMS. The first cured layer of PDMS sets the imaging depth (Fig. 1).

Aneurysm – Prior to completing the steps described above for the straight channel condition, the bare optical fiber was placed in contact with a small drop (~2 μL) of pre-prepared sugar wax that had been poured onto a glass slide. To create the sugar wax, sucrose and 5% acetic acid in water was mixed in a 1:1 ratio and stirred to ensure that the sucrose had been completely dissolved. Using a hot plate, the solution was heated to 115 °C, producing a light amber colored solution. Brass wire of the same diameter can also be used to achieve this geometry in order to avoid deformation of the optical fiber due to the heat of the sugar wax. A small bubble of the sugar wax adhered to the strand of optical fiber and hardened. This step was repeated until the desired aneurysm diameter was achieved, at which point the aneurysm model was cast in PDMS as previously described for the standard channel.

Stenosis – A strand of optical fiber was immobilized on a glass slide with transparent tape. Wrapping the sharp edge of a razor blade with fine-grain (1000 grit) sandpaper enables a precise notch to be “sanded” out of the optical fiber. When the desired degree of stenosis was achieved, the fiber was cast in PDMS as previously described for the standard channel.

Bifurcation – A bifurcation was made by solvent welding 2 strands of optical fiber using a bifurcation glue and custom assembly tool. To prepare the custom bifurcation assembly tool, two glass slides were immobilized parallel to each other with a gap of approximately 1 cm between them in a 100 mm petri dish. This tool can be used as a surface to solvent weld two pieces of optical fiber together. To prepare the bifurcation glue, we dissolved 0.1 g of polystyrene into 500 μL of dichloromethane in a glass vial (under a fume hood). The solution was allowed to sit for 24 hours or until the polystyrene had completely dissolved in the dichloromethane and the solution had noticeably increased in viscosity. Using these materials, a strand of optical fiber was cut at a 45° angle, after which the angled tip was placed in contact to the side of a separate strand of optical fiber over the gap in the bifurcation assembly tool. Both strands were immobilized in a touching position with scotch tape. A 1 μL droplet of bifurcation glue was pipetted on the joined section. The glue was allowed to anneal in a 90 ^o^C oven for 45 minutes in order for the glue to achieve optimal smoothness. The bifurcation model was cast in PDMS as previously described for the standard channel.

### Device Endothelialization

Completed devices were coated with 5% fibronectin (Sigma-Aldrich, St. Louis, MO) and incubated at 37 °C for one hour. Human umbilical vein endothelial cells (HUVECs) (Lonza, Basel, Switzerland) were injected at a seeding density of 1,000,000 cells/mL into the device and incubated at 37 °C for 3 minutes. The devices were rotated 90° from the horizontal axis, refilled with suspended endothelial cells, and incubated at 37 °C for 3 minutes. This rotation process was then repeated 2 more times until endothelial cells evenly coated the channel walls. Endothelialized devices were incubated at 37 °C for four hours while rotating the device about the horizontal axis at 8 rpm using a Hulamixer (Life Technologies, Carlsbad, CA).

An endothelial cell media reservoir was constructed using a 50 mL centrifuge tube with holes drilled into the cap. Thirty-gauge PTFE tubing (Cole-Parmer, Vernon Hills, Illinois) was inserted into each hole until the tubing reached the bottom of the centrifuge tube (at the ~1 mL mark). The joints between the tubing and the holes were sealed with medical adhesive (Dow Corning, Midland, MI) and allowed to dry for 24 hours. Three components were connected in series to achieve proper seeding. First, PTFE tubing (30 gauge) was used to connect the media reservoir to a peristaltic pump to drive the flow. Next, the same tubing was used to connect the pump to the seeded device. Finally, the seeded device was connected back to the media reservoir so that media could be recycled. A 0.2 μm syringe filter (Pall Life Science, Port Washington, New York) was attached to the tubing on the outgoing end of the peristaltic pump to avoid any contamination from the recirculating media (Supplemental Fig 2). EGM-2 media (Lonza, Basel, Switzerland) was infused at 1 mL/min for 24 hours using the closed-loop flow system described above (Supplemental Fig. 5). Additionally, the same seeding protocols were used to seed devices with Human aortic endothelial cells (HAECs) and lung-derived human microvascular endothelial cells (HMVEC-Ls) in order to highlight the versatility of this technique. HMVEC-L cell culture required EGM-2MV media (Lonza, Basel, Switzerland).

### Repeatability Measurements

In order to quantify the repeatability of the device fabrication, we fabricated 5 identical devices of each geometry (Stenosis, Aneurysm, and Bifurcation) and then measured the area of interest for each geometry (degree of stenosis, aneurysm diameter, and angle of bifurcation). Repeatability was reported as the standard deviation of these measurements for each geometry (Fig. 1). We were able to reliably fabricate stenoses to with 4.8%, aneurysm diameter to within 14.5%, and bifurcation to within 3.4%.

### Experiments

VCAM-1 expression – Endothelialized devices were cultured under flow for 24 hours, exposing endothelial cells in the straight cylindrical regions (i.e., not in the regions of the aneurysm, stenosis, or bifurcation) to WSS values of ~10 dynes/cm^2^. Cells were fixed with 4% paraformaldehyde (Electron Microscopy Sciences, Hatfield, Pennsylvania), washed with PBS, and then stained with a mouse polyclonal antibody specific to VCAM-1 (Abcam, Cambridge, UK). Cells were washed again and a secondary goat anti-mouse antibody tagged with Alexa Fluor 488 (Life Technologies, Carlsbad, CA) was added. VCAM-1 expression was visualized via epifluorescence microscopy and compared to wall shear stress values predicted with CFD. Fluorescence intensity was normalized to background fluorescence in all images.

Thrombomodulin expression – Endothelialized devices were cultured under flow for 24 hours, exposing endothelial cells to shear stress of 10 dynes/cm^2^. Cells were fixed with 4% paraformaldehyde (Electron Microscopy Sciences, Hatfield, Pennsylvania), washed with PBS, and then stained with a rabbit monoclonal antibody specific to thrombomodulin (Abcam, Cambridge, UK). Cells were washed again and secondary goat anti-rabbit antibody tagged with Alexa Fluor 488 (Life Technologies, Carlsbad, CA) was added. Thrombomodulin expression was visualized via epifluorescence microscopy and compared with wall shear stress values predicted with CFD. Both VCAM-1 and thrombomodulin expression were quantified with a custom algorithm written in MATLAB that analyzes pixel intensity of fluorescence (which corresponds to molecular expression) in specific regions of interest (ROI) within each geometry and compared to approximate predicted WSS values in the selected ROIs. Fluorescence intensity was normalized to background fluorescence in all images.

VE-Cadherin Expression – Endothelialized devices were cultured under flow for 24 hours, exposing endothelial cells to shear stress of 10 dynes/cm^2^. Endothelial cells were fixed with 4% paraformaldehyde (Electron Microscopy Sciences, Hatfield, Pennsylvania) for 30 min. Fixed endothelial cells were permeabilized with 0.5% Triton – X-100 for 30 minutes. Fixed and permeabilized endothelial cells were stained with a mouse antibody specific to VE-Cadherin (Abcam, Cambridge, UK). A goat anti-mouse secondary antibody tagged with Alexa Fluor 488 (Life Technologies, Carlsbad, CA) was added and VE-Cadherin expression was visualized with via epifluorescence microscopy.

Nitric oxide production – Endothelialized devices were cultured under flow for 24 hours, exposing endothelial cells to shear stress of 10 dynes/cm^2^. Endothelial cells were stained with diaminofluorescein (DAF-2DA, Santa Cruz Biotechnology, Santa Cruz, CA). Nitric oxide production was visualized via epifluorescence microscopy.

Platelet aggregation – Endothelialized stenosis devices were cultured under flow for 24 hours. Human whole blood with CD41 stained platelets in heparin was perfused into endothelialized devices and bare devices at a shear rate of 1800 s^−1^ for 5 minutes. Platelet aggregate size was assessed via epifluorescence microscopy. Platelet staining was conducted using a primary CD41 antibody (Abcam, Cambridge, UK) and an Alexa Fluor 488 secondary antibody.

Red blood cell (RBC) aggregation – A 5% RBC solution in PBS from human healthy control patients or human sickle cell disease patients was perfused through an endothelialized bifurcation device. RBC adhesion to endothelial cells was visualized via epifluorescence microscopy. Whole blood was centrifuged for 15 minutes at 150 g. 100uL of the separated red blood cells from whole blood were suspended in 10 mL of PBS and stained with Rhodamine 18 (Life Technologies, Carlsbad, CA). The RBC solution was incubated at room temperature for 50 minutes. The RBCs were centrifuged for 7 more minutes at 300 g and the excess stain and PBS was removed. Additional experiments were conducted by stimulating the endothelial cells with 20 ng/mL TNF-α in media for 6 hours under the same flow conditions prior to assessing RBC aggregation conditions.

Ultralarge von Willebrand factor (ULVWF) – Endothelialized devices were cultured under flow for 24 hours. Endothelial cells were stimulated with histamine (Sigma-Aldrich, St. Louis, MO) and stained for VWF by infusing (shear 10dynes/cm^2^) a solution containing FITC conjugated anti-VWF primary (Bio-Rad, Hercules, CA) 2% BSA (Sigma-Aldrich, St. Louis, MO), 100 μM histamine (Sigma-Aldrich, St. Louis, MO) in media. ULVWF multimer formation was assessed with confocal microscopy.

All experimental protocols were reviewed and approved by the Emory University Institutional Review Board and carried out in accordance with Emory University IRB 00049877. All human patient samples were obtained with informed consent according to Emory University IRB 00049877.

### Imaging

Confocal Microscopy – LSM 700 (Zeiss, Jena, Germany), CellMask Orange (plasma membrane) (Life Technologies, Carlsbad, CA) and Hoechst (nucleus) (Life Technologies, Carlsbad, CA).

Epiflourescence microscopy – TE2000-u (Nikon, Tokyo, Japan): CellMask orange and Syto-13 (nucleus) (Life Technologies, Carlsbad, CA). Images were taken using either a 4x objective (NA: .13, Nikon, Tokyo, Japan), or a 10x objective (NA: .3, Nikon, Tokyo, Japan). Figure 3h contains 3 images stitched together.

Videos of platelet and RBC aggregation were obtained via epifluorescence microscopy and processed with LabVIEW (National Instruments) frame by frame in a loop, which was executed for the entire length of the video (5 minutes). Using fluorescently labeled RBCs and platelets, a minimum threshold filter, based on fluorescence intensity, was applied to each video frame to identify and locate aggregates. Aggregation size was determined in each frame and plotted versus time.

### Computational Fluid Dynamics Modeling

Devices were stabilized in sample holders of 7 mm OD and scanned using a Scanco uCT50 micro computed tomography (microCT) system. X-ray source, detector, and reconstruction settings: E = 45 kVp, I = 200 μA, 9 W power, 500 ms integration time, 1024 × 1024 pixel matrix with a field of view of 7 mm, yielding voxel size 7 μm. Raw data generated by the scans were automatically reconstructed to 2D slice tomograms using a standard cone beam filtered back projection algorithm. To create binarized 3D images of the casted polymer structures, slice tomograms were stacked and global thresholding was performed based on histogram analysis of intensity values.

Following micro-CT data acquisition, the image volume was exported as a stereolithography file (.stl) for computational domain pre-processing. The 3D point cloud of the lumen surface was extracted, wrapped with a smoothed volume surface, and non-uniform rational B-splines were fit to the surface (Geomagic 11, Geomagic, Inc., Research Triangle, NC). Flow extensions 2 mm in length were added to the inlet and outlet to ensure a smooth transition into the computational domain and fully developed flow at the outlet (ICEM CFD, ANSYS 14, Ansys, Inc.). Note that these flow extensions were shorter than the experimental model; however, because flow fully developed within the extensions prior to arriving at the region of interest (e.g., aneurysm, stenosis, bifurcation), longer flow extensions would not have altered the computational data, but would have required additional computational time. The volume was discretized with an unstructured tetrahedral mesh, which included a boundary layer to capture the near-wall flow patterns (ICEM CFD), and the computational mesh was imported into the commercial CFD solver Fluent (ANSYS 14). Boundary conditions included a steady inlet flow rate, “pressure-free” at the outlet, and no-slip velocity at the rigid wall. The fluid (blood) was assumed to be an incompressible Newtonian fluid with a density and dynamic viscosity of 1.06 g/cm^3^ and 3.5 cP, respectively. Convergence criteria were set to residual errors <10^-5^. Following CFD simulation convergence, the resulting velocity fields were post-processed to quantify the WSS vectors at each nodal surface. Data are presented as color distributions of WSS vector magnitude across the computational domain and spatially averaged within defined regions of interests (e.g., immediately downstream of the stenosis, opposite wall of the aneurysm). Computational results were considered mesh independent when increasing the mesh density by a factor of 2 resulted in changes in WSS values of <4% in the reported regions of interests.

To examine the hemodynamic impact of experimental variations in developed fabrication technique and prescribed inlet flow rates, simulations were performed after systematically and independently adjusting geometric and hemodynamic parameters. Briefly, geometries were virtually altered by increasing or decreasing the parameter of interest (e.g., percent stenosis) in the fabricated vascular geometry by 50% from the original microCT data acquisition (Geomagic 11). Specifically, the severity of the stenosis was altered from approximately 50% stenosis by diameter to 25% and 75%, the average radius of the aneurysm was altered from approximately 0.4 mm to 0.2 and 0.8 mm, and the bifurcation angle was altered from approximately 30° to 15° and 45°. An inlet flow rate of 300 mL/min was prescribed for all original and geometrically modified models. In addition, to investigate the effects of variations in the inlet flow rate on computed WSS values, the flow rate was altered to either 150 mL/min (50% decrease) or 450 mL/min (50% increase) and prescribed at the inlet in the original three manufactured geometries.

## Additional Information

**How to cite this article**: Mannino, R. G. *et al.* “Do-it-yourself *in vitro* vasculature that recapitulates *in vivo* geometries for investigating endothelial-blood cell interactions”. *Sci. Rep.* **5**, 12401; doi: 10.1038/srep12401 (2015).

## Supplementary Material

Supplementary Information
