# Supplementary material for: “Do-it-yourself in vitro vasculature that recapitulates in vivo geometries for investigating endothelial-blood cell interactions”
Source: Sci Rep. 2015 Jul 23;5:12401. doi: 10.1038/srep12401 (PMC4894411; doi:10.1038/srep12401)
Supplement: Supplementary Information [file srep12401-s1.pdf]

Article Title:

"Do-it-yourself *in vitro* vasculature that recapitulates *in vivo* geometries for investigating endothelial-blood cell interactions"

Authors:

Robert G. Mannino, BS[1,2,3], David R. Myers, PhD[1,2,3], Byungwook Ahn, PhD[1,2,3], Yichen Wang[1,2,3], Margo Rollins, MD[1,3], Hope Gole, PhD[1,2], Angela Lin, MS[2], Robert E. Guldberg, PhD[2], Don P. Giddens, PhD[1,2], Lucas H. Timmins, PhD[1,2], Wilbur A. Lam, MD, PhD[*,1,2,3]

Supplemental Figures:

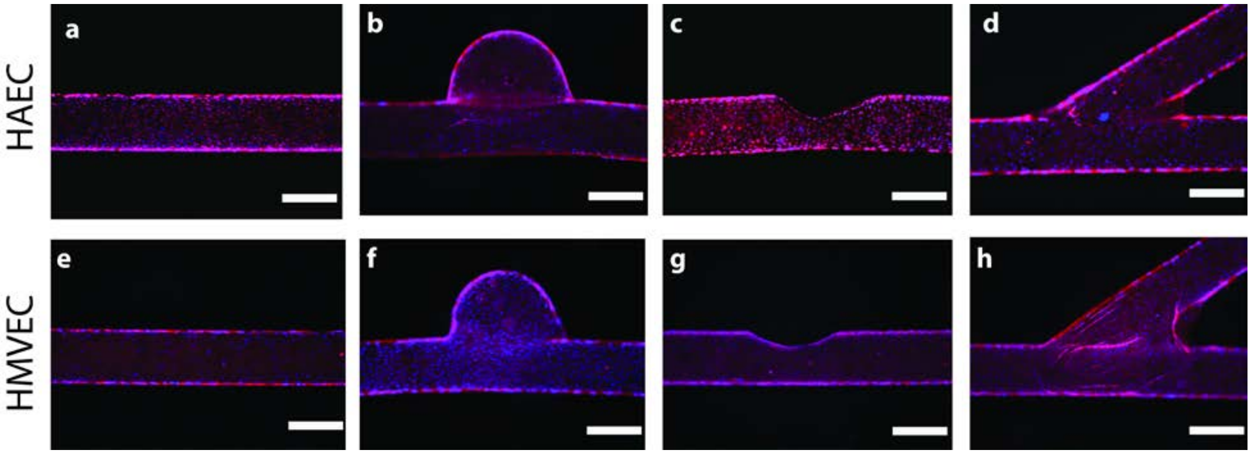

Supplemental Figure 1: The "do-it-yourself" *in vitro* vasculature recapitulates multiple vascular environments. Human aortic endothelial cells (HAEC) (A-D) and lung derived human microvascular endothelial cells (HMVEC) (E-F) can be successfully seeded and grown to confluence within these microchannels in addition to human umbilical vein endothelial cells. Each image represents a separate and unique device seeded with either HAECs or HMVECs. Scale bar is 500µm.

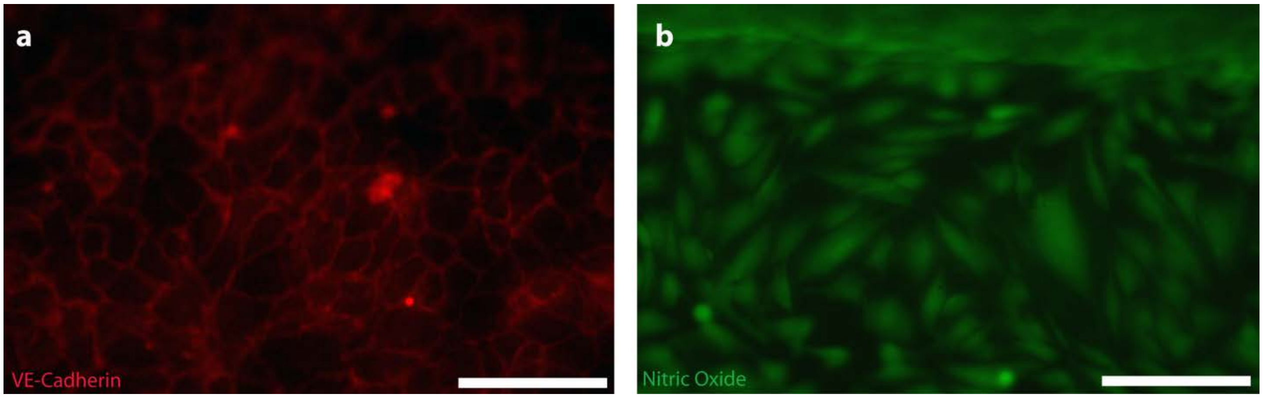

Supplemental Figure 2: Endothelial cells within the "do-it-yourself" vasculature model express VE-cadherin at the cell-cell junctions (A), as well as nitric oxide (B). Scale bar is 250µm.

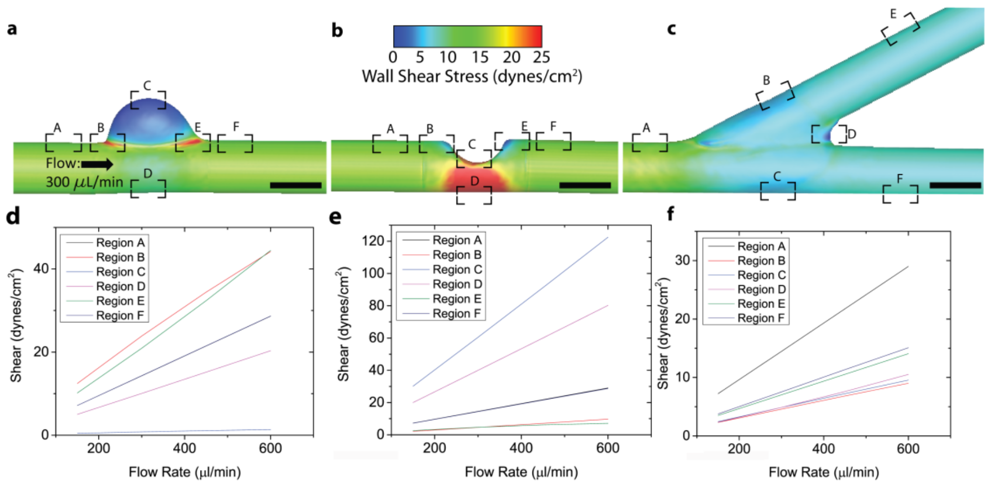

Supplemental Figure 3: Wall shear stress changes with flow rate are approximately linear. Computational fluid dynamics analysis of wall shear stress (WSS) in an A) aneurysm, B) stenosis, and C) bifurcation with highlighted regions of interest (boxed). D-F) Plots displaying WSS vs changing flow rate in each device geometry. Scale bar is 500μm.

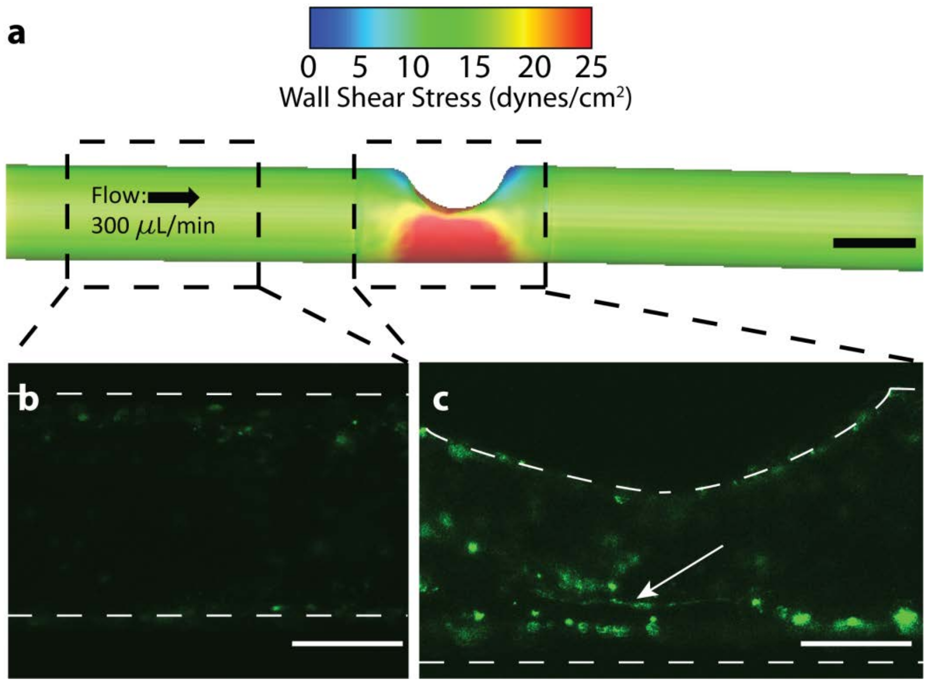

Supplemental figure 4: von Willebrand factor (VWF) preferentially form ultra large von Willebrand factor (ULVWF) polymers in the high wall shear stress (WSS) region of vascular stenosis. A) CFD analysis of WSS in a representative stenosis. B) Fluorescent VWF upstream of the stenosis. C) Fluorescent VWF in the stenotic region. Note the presence of ULVWF strings (arrow). Scale bar is 250μm.

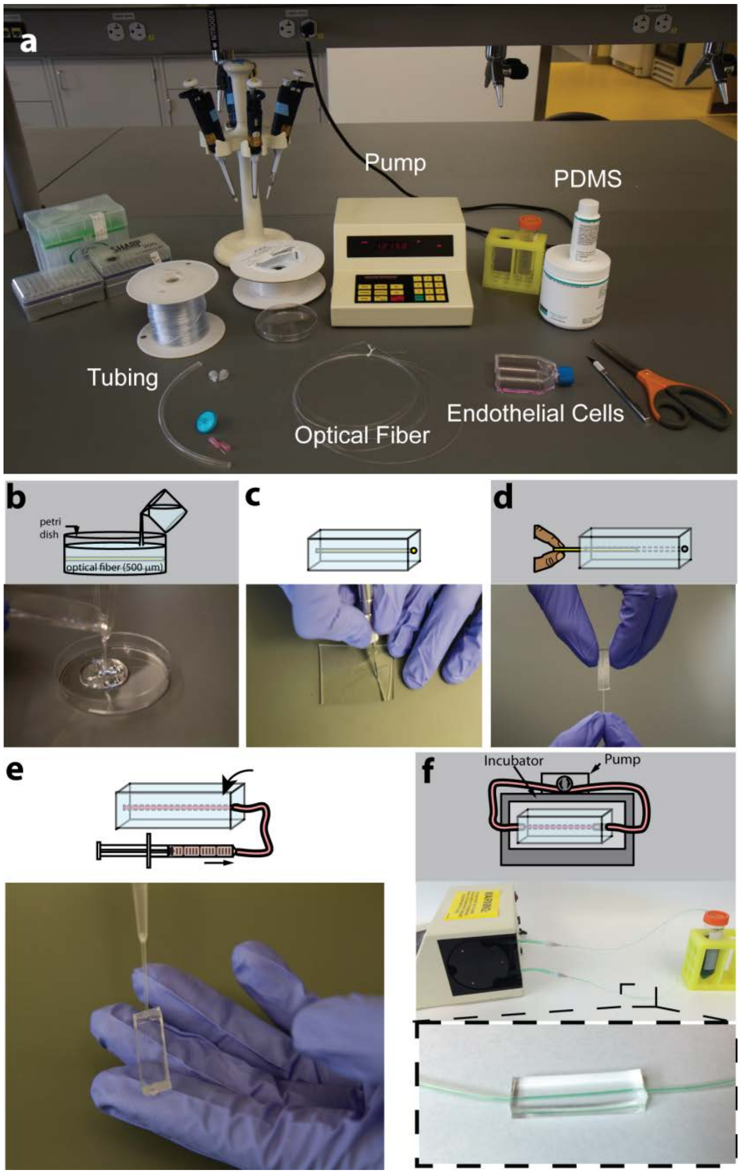

Supplemental Figure 5: Detailed protocol for "do-it-yourself" vasculature fabrication. A) Commonly-used laboratory materials for are used to construct the DIY vasculature. B) PDMS is poured over the optical fiber, encasing and cured. C) Device is cut into desired shape. D) Optical fiber is removed from the PDMS, leaving a hollow flow chamber. E) Endothelial cells are perfused and rotated to achieve uniform seeding. F) The DIY vasculature (Boxed) is connected to a closed flow-loop in a 37°C incubator for cell culture. Drawings in B-F) were prepared by D. R. M and R. G. M.

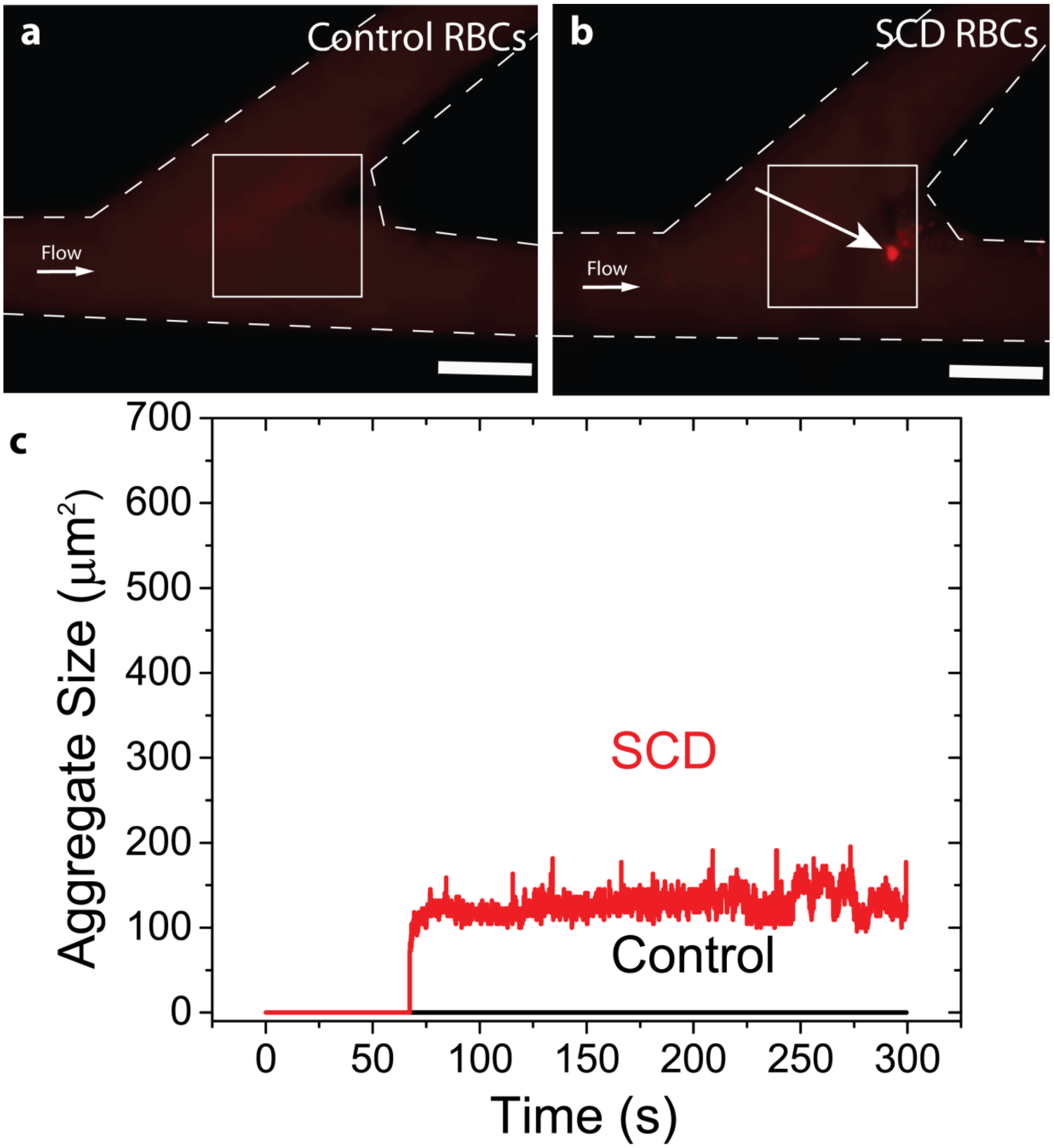

Supplemental Figure 6: Endothelial cell stimulation with tumor necrosis factor- α causes increased aggregation of sickle red blood cells at vascular bifurcations. A) Control RBCs do not form aggregates in a vascular bifurcation after 300 seconds of flow. **B)** SCD RBCs form aggregates (arrow) at vascular bifurcations. **C)** A custom image analysis script (LabVIEW) quantifies the size of RBC aggregates in a region of interest as shown in panels A) and B) in a DIY bifurcation model. Scale bar is 500µm.
